# Identifying Herbal Candidates and Active Ingredients Against Postmenopausal Osteoporosis Using Biased Random Walk on a Multiscale Network

**DOI:** 10.3390/ijms252212322

**Published:** 2024-11-16

**Authors:** Boyun Jang, Youngsoo Kim, Jungbin Song, Young-Woo Kim, Won-Yung Lee

**Affiliations:** 1 IntegroMediLab Co., Ltd., Seoul 04626, Republic of Korea; 2 Department of Herbal Pharmacology, College of Korean Medicine, Kyung Hee University, Seoul 02447, Republic of Korea; 3 School of Korean Medicine, Dongguk University, Gyeongju 38066, Republic of Korea; 4 School of Korean Medicine, Wonkwang University, Iksan 54538, Republic of Korea; 5 Research Center of Traditional Korean Medicine, Wonkwang University, Iksan 54538, Republic of Korea

**Keywords:** postmenopausal osteoporosis, herbs, active ingredients, multiscale network

## Abstract

Postmenopausal osteoporosis is a major global health concern, particularly affecting aging women, and necessitates innovative treatment options. Herbal medicine, with its multi-compound, multi-target characteristics, offers a promising approach for complex diseases. In this study, we applied multiscale network and random walk-based analyses to identify candidate herbs and their active ingredients for postmenopausal osteoporosis, focusing on their underlying mechanisms. A dataset of medicinal herbs, their active ingredients, and protein targets was compiled, and diffusion profiles were calculated to assess the propagation effects. Through correlation analysis, we prioritized herbs based on their relevance to osteoporosis, identifying the top candidates like Benincasae Semen, Glehniae Radix, Corydalis Tuber, and Houttuyniae Herba. Gene Set Enrichment Analysis (GSEA) revealed that the 49 core protein targets of these herbs were significantly associated with pathways related to inflammation, osteoclast differentiation, and estrogen metabolism. Notably, compounds such as falcarindiol from Glehniae Radix and tetrahydrocoptisine from Corydalis Tuber—previously unstudied for osteoporosis—were predicted to interact with inflammation-related proteins, including IL6, IL1B, and TNF, affecting key biological processes like apoptosis and cell proliferation. This study advances the understanding of herbal therapies for osteoporosis and offers a framework for discovering novel therapeutic agents.

## 1. Introduction

Postmenopausal osteoporosis has become a critical global health concern, particularly within aging female populations, where incidence rates continue to rise. This condition, characterized by reduced bone mass and compromised bone microarchitecture, leads to diminished bone strength and an increased risk of fractures. Recent studies have indicated a prevalence of osteoporosis of approximately 23% in women and 11.7% in men, underscoring its disproportionate impact on postmenopausal women [1]. In these women, the sharp decline in estrogen levels disrupts bone remodeling processes by influencing various physiological mechanisms that sustain bone homeostasis. Estrogen deficiency stimulates osteoclast differentiation and activity, accelerating bone resorption to levels that exceed bone formation, thereby decreasing bone density, deteriorating bone structure, and heightening fracture susceptibility [2]. Although the current postmenopausal osteoporosis treatments, including antiresorptive agents like bisphosphonates and selective estrogen receptor modulators (SERMs), and anabolic agents, such as parathyroid hormone (PTH) and teriparatide, provide mechanisms to control bone loss and stimulate bone formation, they exhibit limitations. Efficacy can vary across patient profiles, and these therapies may fail to adequately lower fracture risks in advanced osteoporosis cases. Additionally, long-term bisphosphonate use is associated with potential adverse effects, such as gastrointestinal issues, osteonecrosis of the jaw (ONJ), and atypical fractures, which can impede patient adherence [3,4,5]. Furthermore, the underdiagnosis and undertreatment of osteoporosis in postmenopausal women highlight a significant gap in disease management, necessitating the development of safer and more effective therapeutic strategies to improve long-term outcomes [6].

Herbal medicine offers therapeutic efficacy through its multi-compound composition at low concentrations, allowing for interactions with multiple targets across various biological pathways. This multi-target approach is particularly promising for managing postmenopausal osteoporosis, a complex disorder influenced by hormonal changes and multiple physiological factors. Studies have shown that medicinal herbs and their active compounds can improve bone mineral density and stimulate bone regeneration, highlighting their potential as therapeutic options [7,8]. For instance, clinical trials administering Cimicifugae Rhizoma to postmenopausal women demonstrated reduced bone resorption and increased bone formation without significant side effects [9,10,11]. Additionally, herbs with known anti-osteoporotic properties, such as Sambuci Lignum and Salviae Miltiorrhizae, along with their extracts or active compounds, have effectively prevented bone loss in ovariectomized osteoporosis animal models. These benefits appear to be mediated through mechanisms that promote osteoblast differentiation, regulate osteoclastogenesis, and inhibit collagen degradation [12,13,14,15]. A recent review article further highlighted that diospongins, isolated from Dioscorea spongiosa, exhibit promising biological activities supportive of bone health, including potential anti-osteoporotic effects [16]. Collectively, these findings underscore the therapeutic potential of medicinal herbs for postmenopausal osteoporosis, supporting their development as safe and novel treatment options.

Herbal medicine has long been widely used to manage a range of symptoms and diseases. However, the lack of understanding regarding its molecular mechanisms in the human body limits its development and broader application. Advances in systems biology tools, however, have enabled deeper insights into the mode of action of herbal medicines, particularly for herbs with multi-compound, multi-target properties [17]. Network pharmacology has been instrumental in mapping the complex interactions within biological networks, identifying rational drug targets, and providing a comprehensive overview of disease treatment through interconnected biological pathways. For example, network pharmacology has been used to elucidate the mechanisms of action of Mori Folium (dried leaves of *Morus alba* L.) in diabetes and to identify potential herbal antidepressants, facilitating the selection of an optimal herbal combination for further experimental validation [18,19]. Additionally, the recently proposed multiscale interactome framework, which integrates biological functions and physical protein–protein interactions, provides a robust platform for predicting a drug’s therapeutic potential on specific disease targets and mechanisms. This approach also enables the exploration of new therapeutic applications for existing drugs and supports the identification of active ingredients and the discovery of novel drug candidates [20,21].

**Figure 1:**
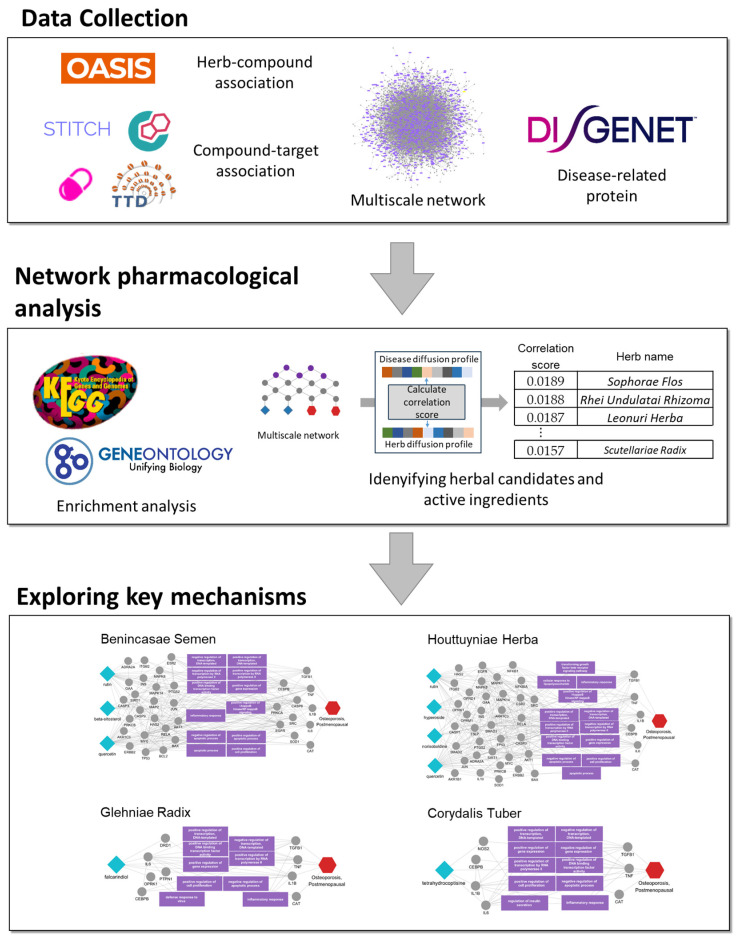
Schematics for identifying candidate herbs and active ingredients for postmenopausal osteoporosis. This schematic illustrates the use of multiscale network analysis to identify herbs and active ingredients with potential efficacy against postmenopausal osteoporosis. Herb–compound and compound–target associations were mapped, and disease-related proteins were identified. Diffusion profiles for herbs and disease proteins were calculated and compared, prioritizing herbs with high correlation scores. Enrichment analysis revealed key biological pathways, while individual ingredients of top herbs were further analyzed to highlight core protein targets. The bottom panel shows network diagrams for selected herbs, indicating relevant protein targets and pathways.

In this study, we applied a network-based approach to identify the candidate herbs and their active ingredients with potential benefits for postmenopausal osteoporosis and to elucidate their potential mechanisms of action (Figure 1). To achieve this, we compiled medicinal herbs, their active ingredients, and associated protein targets. We then calculated diffusion profiles using biased random walks for both herb-specific and disease-related protein targets. By comparing diffusion profiles between the herbs and postmenopausal osteoporosis, we prioritized herbs with high correlation scores, indicating their potential efficacy against the condition, and identified core protein targets and biological functions involved in the proposed treatment. We further investigated the top 10 ranked herbs, assessing the available evidence to support their effectiveness and identify the novel candidate herbs not yet reported for this condition. We further calculated the propagation effects of the individual ingredients of the candidate herbs, prioritizing those with high correlation scores for postmenopausal osteoporosis, and explored the mechanisms of these active ingredients within the multiscale network. Our approach demonstrates the potential of multiscale network analysis for the discovery of novel therapeutic herbs and ingredients and provides a foundation for future research into their therapeutic mechanisms against postmenopausal osteoporosis.

## 2. Results

### 2.1. Identification of Potential Candidate Herbs Against Postmenopausal Osteoporosis

To identify the herbs that are potentially effective against postmenopausal osteoporosis, we first collected herb–ingredient data from the OASIS database. Protein targets associated with these ingredients were then retrieved from validated sources, including DrugBank, TTD, and STITCH. Using this herb target data, postmenopausal osteoporosis-related targets, as well as proteins and biological functions within the multiscale network, we applied a biased random walk algorithm to calculate diffusion profiles. Correlation scores, indicating the similarity between the diffusion profiles of the herb and postmenopausal osteoporosis, were subsequently calculated. Herbs with high correlation scores were identified as promising candidates for postmenopausal osteoporosis treatment. Additionally, a hypergeometric test was conducted to assess the degree of protein overlap.

Herbs with a high correlation score and a significant association (*p*-value < 0.05) with disease-related proteins were prioritized. Among these, the top 10 ranked herbs were selected, specifically those with five or more active ingredients significantly associated with disease-related proteins. An enrichment value of five or higher was also confirmed between these herbs and protein targets related to postmenopausal osteoporosis, indicating that the multiscale network-based prediction model successfully identified targets closely linked to the disease. The results showed that Sophorae Flos had the highest correlation score (0.0189), followed by Rhei Undulatai Rhizoma (0.0188), Leonuri Herba (0.0187), and Benincasae Semen (0.0186), Schizonepetae Spica (0.0174) and Glehniae Radix (0.0173), each exhibiting a high correlation coefficient (Table 1).

Among the top 10 ranked herbs, Sophorae Flos, Rhei Undulatai Rhizoma, Leonuri Herba, Schizonepetae Spica, Cnidi Fructus, and Anemarrhenae Rhizoma have been previously reported to benefit postmenopausal osteoporosis [22,23,24,25,26,27,28,29,30], indicating that our predictions effectively align with findings from the earlier studies. For Sophorae Flos, both its extract and the ingredient sophoridine have demonstrated the potential to treat osteoporosis by inhibiting osteoclast differentiation in estrogen-deficient animal models induced by ovariectomy [22,23]. Leonuri Herba, commonly used for female-related conditions, has been shown to support bone health by promoting osteoblast differentiation and inhibiting osteoclast formation [25,26]. Schizonepetae Spica exhibited protective effects in inflammation-induced bone loss models by reducing osteoclast formation and activity through the suppression of Akt and IkB phosphorylation [27]. Research on Cnidi Fructus primarily focused on its anti-osteoporotic properties, with particular emphasis on its key ingredient, osthole, which is considered the most promising compound for further study [28,29]. In contrast, Benincasae Semen, Glehniae Radix, Corydalis Tuber, and Houttuyniae Herba have limited or no prior evidence supporting their use for postmenopausal osteoporosis treatment. In this study, however, these herbs showed high correlation scores, and significant protein overlap with the postmenopausal osteoporosis diffusion profile. These findings suggest that these herbs could serve as promising novel candidates for therapeutic strategies for postmenopausal osteoporosis.

### 2.2. Herb–Ingredient–Target Network Construction of the Top 10 Herbs

**Figure 2:**
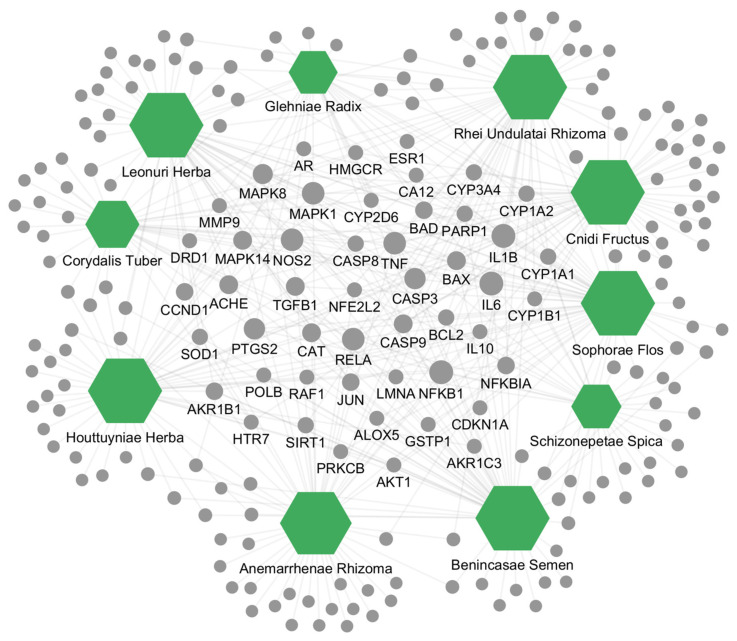
Herb–target interaction network of the top 10 candidate herbs with high correlation scores for postmenopausal osteoporosis. Green hexagons represent herbs, and gray circles represent protein targets. Edges indicate interactions between herbs and targets, with the size of hexagons and circles reflecting interaction frequency (ranging from 1 to 50). Names of the 49 core protein targets, targeted by three or more of the 10 herbs, are displayed.

Subsequently, we constructed and visualized an interaction network to map the relationships among the top 10 ranked herbs, identified as having high correlation scores with postmenopausal osteoporosis, and their respective protein targets using Cytoscape 3.10.2 (Figure 2). This network comprises 443 interactions (edges) between 10 herbs and 210 targets, effectively illustrating the multi-target nature of multi-component herbs and their potential therapeutic effects through complex biological interactions. Of these targets, only 49 proteins were targeted by 3 or more of the 10 herbs, underscoring their potential role as core protein targets. Notably, IL6, IL1B, and NFKB1 were common targets across all 10 herbs, while TNF, MAPK1, RELA, and NOS2 were each targeted by 9 herbs. These findings suggest that these core protein targets have a pivotal role in the mechanisms of action of these herbs and are likely critical in the treatment of postmenopausal osteoporosis.

### 2.3. Gene Set Enrichment Analysis (GSEA) of the Top 10 Herbs

**Figure 3:**
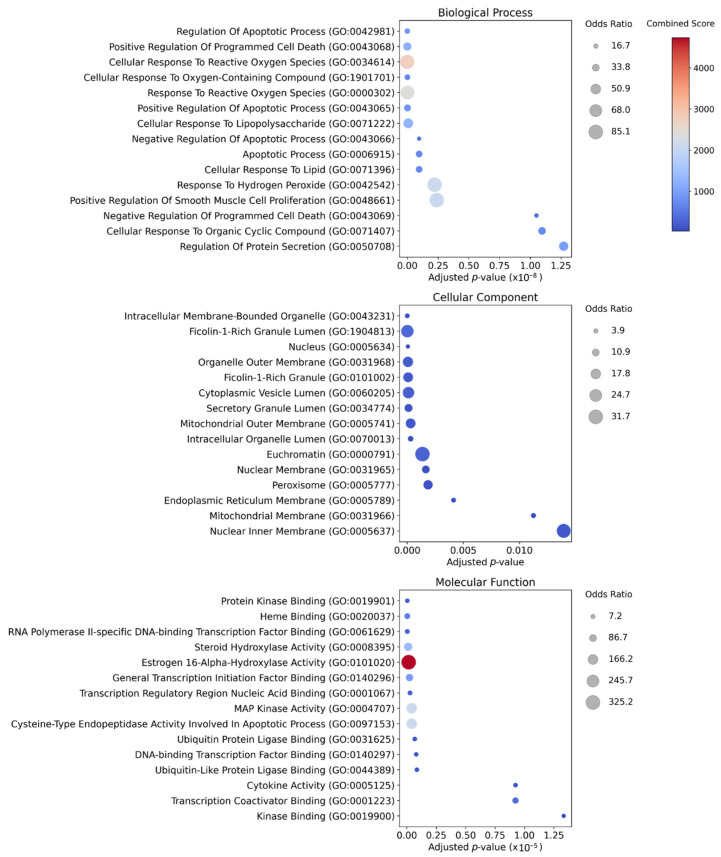
Gene Ontology Enrichment Analysis for Core Protein Targets. Gene Ontology enrichment analysis of the 49 core protein targets across three categories: biological processes (**top**), cellular components (**middle**), and molecular functions (**bottom**). The x-axis represents the adjusted *p*-value (indicating the association significance), bubble size corresponds to the odds ratio, and bubble color reflects the combined score, which indicates the statistical significance of each term. Visualization highlights the most significantly enriched terms across each category.

To further investigate these core protein targets of herbs, we performed Gene Set Enrichment Analysis (GSEA) using KEGG and Gene Ontology to identify the signaling pathways and biological functions associated with the selected top 10 herbs. KEGG analysis highlighted significant associations with several critical pathways, including the AGE–RAGE signaling pathway, apoptosis, and C-type lectin receptor signaling pathway. Additional pathways related to inflammatory cytokine activation, such as the TNF and IL-17 signaling pathways, as well as osteoclast differentiation, ranked among the top 15 pathways (Table 2). Gene Ontology analysis revealed strong associations with essential biological functions, primarily those involved in the regulation of apoptosis and response to reactive oxygen species. Notably, inflammation-related cellular responses to lipopolysaccharides and lipids also demonstrated significant associations (Figure 3, top). Further analysis of cellular component locations for these core protein targets showed a predominant localization in the mitochondria, nucleus, cytoplasmic vesicles, secretory granules, and peroxisomes, which are organelles critical for cell survival, cellular signal transduction, and bone cell homeostasis (Figure 3, middle). Notably, in the molecular function category of Gene Ontology analysis, estrogen 16-alpha-hydroxylase activity demonstrated the highest combined score, indicating a strong association. This suggests that the core protein targets are significantly involved in estrogen metabolism, a primary factor in postmenopausal osteoporosis, and thus influence osteoporosis progression (Figure 3, bottom). These findings imply that the central role of these core protein targets in regulating molecular signaling pathways and biological functions provides a strong basis for their potential application in therapeutic strategies for postmenopausal osteoporosis.

### 2.4. Identification of Potential Active Ingredients and Multiscale Network Mechanism Analysis of Candidate Herbs

We performed an additional analysis of the ingredients and multiscale network mechanisms of the candidate herbs, Benincasae Semen, Glehniae Radix, Corydalis Tuber, and Houttuyniae Herba, identified as potential treatments for postmenopausal osteoporosis. For each herb, our objective was to predict therapeutic mechanisms through which their ingredients might influence postmenopausal osteoporosis. Using target data for each ingredient, we applied multiscale network analysis to calculate correlation scores, evaluating each ingredient’s potential impact on postmenopausal osteoporosis. Ingredients with high correlation scores and significant protein overlap were prioritized as active ingredients.

This analysis identified rutin (0.0217), beta-sitosterol (0.0161), and quercetin (0.0092) as the active ingredients in Benincasae Semen, each exhibiting high correlation scores with the postmenopausal osteoporosis diffusion profile. In Houttuyniae Herba, norisoboldine (0.0473), hyperoside (0.0261), rutin (0.0217), and quercetin (0.0092) showed significant correlations with postmenopausal osteoporosis-related targets. Glehniae Radix was characterized by falcarindiol (0.0557), and Corydalis Tuber by tetrahydrocoptisine (0.0846), each as the sole ingredient with both high correlation scores and statistically significant protein overlap within the postmenopausal osteoporosis diffusion profile (Table 3).

Most of the prioritized active ingredients, particularly those derived from Benincasae Semen and Houttuyniae Herba, have documented therapeutic efficacy in postmenopausal osteoporosis. In contrast, falcarindiol from Glehniae Radix and tetrahydrocoptisine from Corydalis Tuber have no prior evidence of efficacy for this condition, suggesting that these ingredients may represent novel active ingredients with potential benefits for postmenopausal osteoporosis. These findings indicate that our multiscale network approach successfully prioritized active ingredients with known or potential efficacy against postmenopausal osteoporosis.

**Figure 4:**
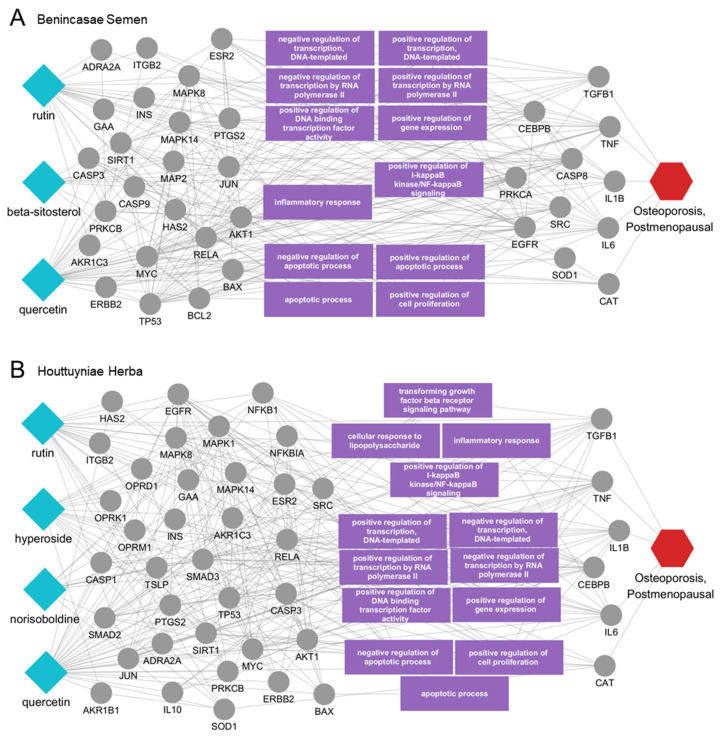
Predicted multiscale network mechanisms of active ingredients from candidate herbs. (**A**) Rutin, beta-sitosterol, and quercetin from Benincasae Semen, (**B**) Rutin, hyperoside, norisoboldine, and quercetin from Houttuyniae Herba. Light blue diamond shapes indicate the active ingredients, gray circles represent the protein targets, and the red hexagon denotes the disease of interest. Purple rectangles highlight distinct biological functions associated with the targets.

To explore the key mechanisms underlying the activity of these active ingredients, we constructed a subnetwork comprising protein targets and biological functions significantly influenced by postmenopausal osteoporosis and these active ingredients. The constructed network for Benincasae Semen identified that rutin, beta-sitosterol, and quercetin directly interacted with disease-related proteins, including TGFB1, TNF, IL1B, IL6 and CAT. Other proteins were involved in disease-associated biological functions or were indirectly linked to disease-related proteins. The key biological functions represented included the regulation of gene expression, inflammatory response, NF-kappaB signaling, and apoptosis, all of which are associated with postmenopausal osteoporosis (Figure 4A).

Similarly, the analysis visualized the direct interactions of norisoboldine, hyperoside, rutin, and quercetin from Houttuyniae Herba with TGFB1, TNF, IL1B, IL6, and CAT. These interactions, both direct and indirect, influenced biological functions such as the TGF-beta receptor signaling pathway, cellular response to lipopolysaccharide, inflammatory response, NF-kappaB signaling, gene expression regulation, and apoptotic process (Figure 4B).

**Figure 5:**
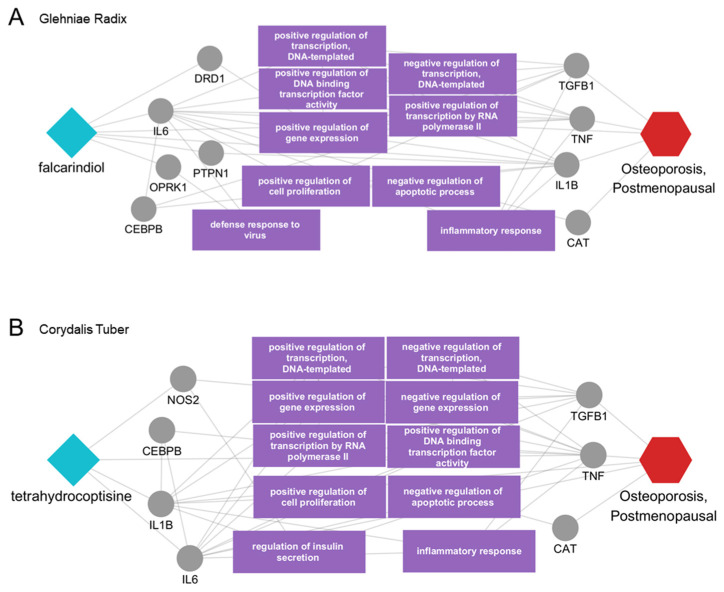
Predicted multiscale network mechanisms of falcarindiol and tetrahydrocoptisine. (**A**) falcarindiol from Glehniae Radix, (**B**) tetrahydrocoptisine from Corydalis Tuber. Light blue diamond shapes indicate the active ingredients, gray circles represent the protein targets, and the red hexagon denotes the disease of interest. Purple rectangles highlight distinct biological functions associated with the targets.

The multiscale network mechanisms of the novel active ingredients, previously lacking evidence, were further investigated to predict their molecular interactions that may underpin their therapeutic effects. The network indicated that falcarindiol from Glehniae Radix directly affected disease-related proteins, including IL6, IL1B, and TNF. Additionally, falcarindiol interacted with other proteins such as DRD1, PTPN1, and OPRK1, which are engaged in a network of interactions with other proteins linked to the disease. These proteins are involved in regulating gene expression, transcriptional activity, and key cellular processes, including apoptosis, cell proliferation, and inflammatory responses (Figure 5A). Next, the impact of tetrahydrocoptisine, an active ingredient in Corydalis Tuber, on disease-related targets was visualized (Figure 5B). Tetrahydrocoptisine was found to modulate postmenopausal osteoporosis by directly interacting with disease-related proteins such as IL6, IL1B, and TNF, in addition to affecting disease-associated biological functions and indirectly interacting with other disease-related proteins. The findings further suggested that the therapeutic effect of tetrahydrocoptisine was associated with the regulation of gene expression, apoptosis, cell proliferation, and inflammation. In summary, this study highlights the therapeutic potential of active ingredients from candidate herbs for postmenopausal osteoporosis and provides valuable mechanistic insights by predicting the biological functions of the proteins involved in their therapeutic effects.

## 3. Discussion

This study employed a multiscale network and random walk-based analysis to identify the potential candidate herbs with therapeutic effects on postmenopausal osteoporosis and to elucidate their mechanisms of action. The top 10 ranked herbs, which showed high correlation scores and statistically significant associations (*p*-value < 0.05) within the disease-related protein network, included Sophorae Flos, Rhei Undulatai Rhizoma, Leonuri Herba, Benincasae Semen, Schizonepetae Spica, Glehniae Radix, Cnidi Fructus, Anemarrhenae Rhizoma, Corydalis Tuber, and Houttuyniae Herba. Notably, some herbs with limited or no prior evidence of efficacy for postmenopausal osteoporosis, such as Benincasae Semen, Glehniae Radix, Corydalis Tuber, and Houttuyniae Herba, were identified as novel candidate herbs. Meanwhile, herbs with previously reported efficacy, including Sophorae Flos, Rhei Undulatai Rhizoma, Leonuri Herba, Schizonepetae Spica, Cnidi Fructus and Anemarrhenae Rhizoma, further supported the robustness of the network pharmacology approach utilized in this study. Overall, these findings enhance our understanding of the effects of multi-compound traditional herbs on postmenopausal osteoporosis and provide a foundation for the discovery of new therapeutic agents.

The multiscale network analysis used in this study offered a powerful approach for assessing the broad impacts of therapeutics and diseases within the human interactome, accounting for the complex biological functions and pathways beyond protein–protein interactions [31]. Previous research has shown that this approach outperforms other network-based methods in identifying active herbal ingredients and their therapeutic effects on diseases [32]. By simulating propagation effects within the network and calculating interaction similarities using a biased random walk algorithm, prediction accuracy was enhanced. This was achieved by adjusting the transition probabilities to favor movements toward biological functions over individual proteins. As a result, this approach provided a comprehensive view of how the protein targets of herbs or active ingredients influence the critical biological pathways and mechanisms. Previously applied to predict the therapeutic effects of polyphenols in oxidative liver damage [32], this method was extended here to postmenopausal osteoporosis. The approach successfully identified candidate herbs and their core protein targets, which impact disease-associated proteins and pathways in postmenopausal osteoporosis.

Using Gene Set Enrichment Analysis (GSEA) with KEGG and Gene Ontology, we identified the signaling pathways and biological functions linked to the core protein targets of our candidate herbs that are potentially effective against postmenopausal osteoporosis. The KEGG analysis result revealed that pathways related to inflammation and cell survival, such as the AGE–RAGE signaling pathway, apoptosis, and C-type lectin receptor signaling, play central roles in the therapeutic potential of these herbs. For example, the abnormal accumulation of advanced glycation end products (AGEs) on collagen, often linked to diabetes, activates the AGE–RAGE signaling pathway. This activation leads to the inhibition of osteoblast differentiation and the enhancement of osteoclast-mediated bone resorption, thereby contributing to bone loss [33,34], while C-type lectin receptor signaling modulates osteoporosis progression by regulating osteoclast activity and influencing the immune responses within bone tissue [35]. Moreover, the presence of signaling pathways related to inflammatory cytokines, such as TNF and IL-17, among the top ranked target-associated pathways highlighted the significant impact of inflammation on bone metabolism in postmenopausal osteoporosis [36]. The identification of osteoclast differentiation, a primary pathological mechanism of osteoporosis, further clarified the role of osteoclast activation in bone loss after menopause [2]. Pathways involved in immune response regulation, such as the Toll-like receptor and NOD-like receptor signaling pathways, were also identified. Moreover, the FoxO and Sphingolipid signaling pathways, which are associated with cell survival and oxidative stress regulation, suggested a potential role in maintaining bone cell survival and homeostasis [37,38]. Gene Ontology (GO) analysis further revealed that the targets of candidate herbs are significantly associated with biological functions related to apoptosis regulation, oxidative stress response, and inflammation which are major factors in bone cell survival and the progression of postmenopausal osteoporosis [39]. Associations were also found with cellular responses to lipopolysaccharides and lipids, which are closely linked to the inflammatory pathways highlighted the KEGG analysis. In the molecular function category, a strong association was found with estrogen 16-alpha-hydroxylase activity. This enzyme converts estrogen to 16α-hydroxyestrone (16α-(OH)E1), a metabolite known for its estrogen-like physiological effects, particularly in supporting bone formation [40]. Clinical studies on patients with postmenopausal bone loss have shown that decreased activity of estrogen 16-alpha-hydroxylase may reduce estrogen’s bone-protective effects thus accelerating bone loss [41]. Collectively, these findings suggest that the therapeutic effects of candidate herbs with high correlation scores for postmenopausal osteoporosis may be mediated through core targets involved in various signaling pathways and biological functions known to influence the disease.

Our study identified novel candidate herbs, including Benincasae Semen, Glehniae Radix, Corydalis Tuber, and Houttuyniae Herba, which previously lacked clear evidence of efficacy in treating postmenopausal osteoporosis. Benincasae Semen demonstrated anti-inflammatory, nephroprotective, cytotoxic and anti-cancer effects, while Glehniae Radix possesses antioxidant, antitussive, and immune-regulating properties [42,43]. Corydalis Tuber is known for its analgesic and blood circulation promoting effects [44]. Houttuyniae Herba has a broad pharmacological profile, including antioxidant, anti-inflammatory, anti-cancer, anti-bacterial and hepatoprotective effects, with emerging interest in its skincare applications [45,46]. These therapeutic profiles support further exploration of these herbs’ bioactive compounds as candidates for expanded health applications, including postmenopausal osteoporosis. Our multiscale network analysis further demonstrated that active ingredients in these candidate herbs exhibit high correlation scores as well as significant protein overlap with postmenopausal osteoporosis-related targets, indicating potential therapeutic relevance. The constructed subnetworks for Benincasae Semen and Houttuyniae Herba elucidated the mechanisms through which these active ingredients may act on postmenopausal osteoporosis. The direct interactions of rutin, beta-sitosterol, quercetin, norisoboldine, and hyperoside with key disease-related proteins such as TGFB1, TNF, IL1B, IL6, and CAT suggested that these compounds may modulate critical pathways involved in the pathology of postmenopausal osteoporosis. These interactions influenced essential biological functions, including the regulation of gene expression, inflammatory response, NF-kappaB signaling, and apoptosis.

Notably, falcarindiol from Glehniae Radix and tetrahydrocoptisine from Corydalis Tuber, have not been previously reported in association with postmenopausal osteoporosis. Our analysis predicted that these compounds modulate key disease-associated biological functions, including inflammatory response, apoptosis, and cell proliferation. Both falcarindiol and tetrahydrocoptisine demonstrated direct interaction with pro-inflammatory cytokines IL6, IL1B, and TNF, key drivers of osteoclastogenesis and bone resorption in postmenopausal osteoporosis by activating inflammatory pathways linked to bone loss [36]. Additionally, these compounds were found to influence gene expression and bone cell survival through indirect interactions with proteins such as DRD1, CEBPB, OPRK1, PTPN1, NOS2, TGFB1, and CAT. For example, CEBPB (C/EBPβ), a transcription factor involved in the differentiation of osteoblasts and adipocytes, modulates the balance between these cells and regulates gene expression relevant to bone formation [47]. Alterations in TGF-β1(TGFB1), as a key regulator of bone formation and resorption balance, influenced by genetic polymorphisms and estrogen deficiency are known to increase the risk of postmenopausal osteoporosis, highlighting the potential relevance of this interaction in mitigating disease progression [48,49,50]. Although falcarindiol, also known as (3R,8S)-falcarindiol, has been identified as a structural component of phthalides in Angelica Radix with known anti-osteoporotic activity, its role as a constituent of Glehniae Radix remains unexplored [51]. Collectively, these findings underscore the potential of these herbal compounds as novel candidates for postmenopausal osteoporosis treatment and provide valuable insights into their mechanisms of action and associated protein functions, establishing a foundation for future research and clinical applications.

Despite these findings, our study has several limitations that warrant consideration for future research. First, our method for predicting key protein targets of the herbs was based on the hypothesis of overlapping effects within the network, which might require additional optimization to improve predictive accuracy. Second, while our therapeutic effect predictions relied on a multiscale network analysis, we did not account for specific mechanisms of action, such as inhibition or activation, between the compounds and their targets. This gap suggests the need for further validation studies to elucidate these interactions. Additionally, the disease-related proteins were identified from a single database, potentially limiting the comprehensiveness of our analysis. Nevertheless, to the best of our knowledge, this study is the first to systematically identify herbal candidates and active ingredients for postmenopausal osteoporosis using a biased random walk on a multiscale network, providing a valuable foundation for future investigations.

## 4. Materials and Methods

### 4.1. Herb–Ingredient–Target Network Construction

Herbs and their ingredient data were retrieved from the OASIS traditional medicine database (https://oasis.kiom.re.kr/index.jsp, (accessed on 21 August 2024)), managed by the Korean Institute of Oriental Medicine (KIOM). The OASIS platform provides data on potential active ingredients extracted from herbs, identified through physicochemical analysis techniques such as HPLC and UPLC and validated by pharmacological and traditional medicine experts. In this study, we collected 12,871 associations between 420 herbs and 4786 ingredients, each identified through its PubChem CID. These ingredient data served as foundation input for the subsequent network analysis. Ingredient–target interaction data, that had been experimentally validated, were compiled from reputable databases including DrugBank 5.0 [52], Therapeutic Target Database (TTD 2.0) [53], and the Search Tool for Interactions of Chemicals (STITCH 5) [54], which offer comprehensive information on established and potential targets, associated diseases, biological pathways, and drugs targeting these proteins. For precise target identification, SynGO 1.2 was used to align gene symbols and Uniprot IDs with Entrez Gene IDs [55].

A network was then constructed to represent the associations between herbs, ingredients, and protein targets. In this network, nodes represent the following three types of entities: herbs, ingredients, and protein targets. Edges represent the relationships between these entities, specifically herb–ingredient associations and ingredient–target interactions. All edges were unweighted and undirected, indicating the presence of an association or interaction without implying directionality or strength. The ingredients identified by PubChem CID were compared and integrated with the ingredient–target data. Herbs containing fewer than three target-associated ingredients were excluded to ensure sufficient data for reliable network analysis. This threshold was chosen because herbs with at least three active components are more likely to exhibit meaningful pharmacological effects and provide robust interaction data, which enhances the reliability of our predictions. The resulting network enabled the visualization and analysis of interactions among the herbs, components, and protein targets. In this network analysis, the simple pathway count for each herb was calculated, accounting for instances where multiple components influenced a single target. This process allowed for the selection of the top 50 targets, with each target’s relative importance assessed accordingly.

### 4.2. Gene Set Enrichment Analysis (GSEA)

Biological processes and signaling pathways associated with the protein targets were identified using gene set enrichment analysis (GSEA) with the GSEApy module (version 1.1.3) in a Python 3.7 environment, facilitated through the Enrichr platform (http://amp.pharm.mssm.edu/Enrichr/, (accessed on 12 September 2024)) [56,57]. Enrichr performs enrichment analysis by drawing on various gene-set libraries, such as Gene Ontology and the Kyoto Encyclopedia of Genes and Genomes (KEGG) [58,59]. In this study, adjusted *p*-values, z-scores, and combined scores were calculated to evaluate the signaling pathways and biological functions relevant to herbal ingredient targets. The combined score, multiplying the logarithm of the *p*-value with the z-score, provided reliable results, allowing for a systematic evaluation of the effects of herbal components on specific biological pathways. All signaling pathways identified through enrichment analysis were included in the analysis, except for those specifically related to diseases.

### 4.3. Disease-Related Targets

In this study, we utilized postmenopausal osteoporosis-related protein data curated by Ruiz et al. to analyze the proteins associated with postmenopausal osteoporosis [20]. This dataset was sourced from DisGeNet 2019 (https://www.disgenet.org/, (accessed on 12 September 2024)), a database that rigorously maps disease–gene associations to ensure research reliability [60]. We focused solely on expert-curated disease–gene associations provided by DisGeNet for “Osteoporosis, Postmenopausal” thus ensuring relevance to our study. Data in this curated set draw from highly regarded sources, including UniProt, the Comparative Toxicogenomics Database, Orphanet, Clinical Genome Resource, Genomics England PanelApp, Cancer Genome Interpreter, and the Psychiatric Disorders Gene Association Network. We excluded disease–gene associations based on homology in animal models or derived from computational literature mining, as well as associations labeled as therapeutic. This refined disease–protein interaction network formed the basis for validating the protein information related to postmenopausal osteoporosis in our analysis.

### 4.4. Multiscale Network Analysis for Predicting Disease Associations

The multiscale interactome was constructed following the methodology of Ruiz et al., integrating the following three interaction types: protein–protein, protein–biological function, and biological function–function interactions [20]. Human protein–protein interaction data were sourced from the Biological General Repository for Interaction Datasets (BioGRID 3.5.178), the Database of Interacting Proteins (DIP), and the Human Reference Protein Interactome Mapping Project (HuRI), encompassing 387,626 physical interactions among 17,660 proteins. Protein–biological function interactions were derived from the human Gene Ontology database, assembling 34,777 experimentally verified associations between 7993 proteins and 6387 biological functions. Finally, biological function–function interactions were organized into a highly interconnected hierarchical structure, with 22,545 associations among 9798 functions.

Diffusion profiles were then calculated using the multiscale interactome to assess the propagation effects between the herbal ingredients and proteins associated with postmenopausal osteoporosis. This analysis utilized a biased random walk with a restart algorithm, enabling a quantitative evaluation of the influence exerted by herbal targets and ingredient targets within herbs on postmenopausal osteoporosis-related proteins. Correlation score was then calculated between herb–ingredient and disease profiles, facilitating the identification of potential candidate herbs and ingredients for treating postmenopausal osteoporosis.

The primary mechanisms of each ingredient–disease pair were identified by analyzing diffusion profiles and selecting the top k-proteins or biological functions based on their influence from either the drug or the disease. A network was then constructed from these selected entities to highlight their significance. Targets of ingredients that were not associated with disease-related proteins or biological functions were excluded. The highest-ranking entity in the diffusion profile was deemed the most essential for treatment due to its considerable influence. In our analysis, we set the value of k to 20 to ensure sufficient exploration of influential nodes. A previous study indicated that when k was set to 10, the top nodes accounted for approximately 50% of the total visitation frequency in the diffusion profile. By increasing k to 20, we were able to capture a larger portion of the visitation frequency, enhancing the comprehensiveness of our analysis. For a comprehensive explanation of the diffusion profile calculation method, including specific mathematical formulas, iterative processes, and the rationale for selecting parameter k, please refer to the prior research [20].

## 5. Conclusions

In conclusion, this study demonstrated a comprehensive multiscale network analysis approach to proposing novel herbs and compounds for the treatment of postmenopausal osteoporosis. This methodology enabled the identification of promising therapeutic candidates by exploring complex protein interactions and biological pathways, including previously understudied herbs. However, a limitation of this predictive approach is its inability to specify precise modes of action, such as activation or inhibition. To address this, validation of the predicted outcomes through in vitro, in vivo, and clinical studies is essential. Additionally, as database reliability and scope affect prediction accuracy, integrating the latest data and diverse sources remains a critical objective. Despite these limitations, this study presents a pioneering strategy for developing treatments for postmenopausal osteoporosis, combining multiscale network and random walk algorithms. It holds substantial academic value by introducing an innovative methodological framework for future medicinal herb research.

## Figures and Tables

**Table 1:** Top 10 ranked herbs identified strong correlation with postmenopausal osteoporosis.

Herb Name (Latin)	Correlation Score	Overlap (*p*-Value ^#^)	Enrichment	References (PMID)
Sophorae Flos	0.0189	5/50 (3.51 × 10^−11^)	118.36	29058425, 39104339
Rhei Undulatai Rhizoma	0.0188	5/50 (3.51 × 10^−11^)	118.36	29693149
Leonuri Herba	0.0187	5/50 (3.51 × 10^−11^)	118.36	31524244, 33708763, 30224063, 31328430
Benincasae Semen *	0.0186	5/50 (3.51 × 10^−11^)	118.36	-
Schizonepetae Spica	0.0174	3/31 (1.29 × 10^−6^)	114.54	27550314
Glehniae Radix *	0.0173	3/30 (1.17 × 10^−6^)	118.36	-
Cnidi Fructus	0.0163	4/50 (2.24 × 10^−8^)	94.69	38507853, 31081953
Anemarrhenae Rhizoma	0.0162	4/48 (1.89 × 10^−8^)	98.63	16723092, 30272269
Corydalis Tuber *	0.0160	3/34 (1.72 × 10^−6^)	104.44	-
Houttuyniae Herba *	0.0157	4/50 (2.24 × 10^−8^)	94.69	-

The ^#^ symbol next to the *p*-value indicates values obtained using the hypergeometric test, applied to evaluate the significance of overlap between datasets. An * next to the herb name marks candidate herbs strongly associated with postmenopausal osteoporosis that have not yet been investigated.

**Table 2:** KEGG Signaling Pathway Enrichment Analysis of Core Protein Targets.

Term	Overlap	Adjusted *p*-Value	Combined Score	Genes
AGE-RAGE signaling pathway in diabetic complications	16/100	4.48 × 10^−24^	6573.87	*JUN*;*TGFB1*;*PRKCB*;*MAPK14*;*TNF*;*RELA*;*NFKB1*;*IL6*;*MAPK8*;*CCND1*; *IL1B*;*CASP3*;*BCL2*;*BAX*;*AKT1*;*MAPK1*
Apoptosis	17/142	1.79 × 10^−23^	4700.81	*JUN*;*PARP1*;*BAD*;*TNF*;*RELA*;*NFKB1*;*NFKBIA*; *CASP9*;*MAPK8*;*CASP8*;*CASP3*;*LMNA*;*BCL2*;*BAX*;*AKT1*;*MAPK1*;*RAF1*
C-type lectin receptor signaling pathway	15/104	6.15 × 10^−22^	5119.68	*IL10*;*JUN*;*MAPK14*;*PTGS2*;*TNF*;*RELA*;*NFKB1*;*NFKBIA*; *IL6*;*MAPK8*;*CASP8*;*IL1B*;*AKT1*;*MAPK1*;*RAF1*
TNF signaling pathway	15/112	1.64 × 10^−21^	4589.15	*JUN*;*MAPK14*;*PTGS2*;*MMP9*;*TNF*;*RELA*;*NFKB1*; *NFKBIA*;*IL6*;*MAPK8*;*CASP8*;*IL1B*;*CASP3*;*AKT1*;*MAPK1*
IL-17 signaling pathway	14/94	8.35 × 10^−21^	4863.44	*JUN*;*MAPK14*;*PTGS2*;*MMP9*;*TNF*;*RELA*;*NFKB1*; *NFKBIA*;*IL6*;*MAPK8*;*CASP8*;*IL1B*;*CASP3*;*MAPK1*
Pathways of neurodegeneration	20/475	3.31 × 10^−19^	1325.74	*NOS2*;*BAD*;*PRKCB*;*MAPK14*;*PTGS2*;*TNF*;*RELA*;*NFKB1*;*SOD1*;*CASP9*; *IL6*;*MAPK8*;*CASP8*;*IL1B*;*CASP3*;*CAT*;*BCL2*;*BAX*;*MAPK1*;*RAF1*
Toll-like receptor signaling pathway	12/104	1.21 × 10^−16^	2707.61	*NFKBIA*;*JUN*;*IL6*;*MAPK8*;*CASP8*;*IL1B*;*AKT1*; *MAPK1*;*MAPK14*;*TNF*;*RELA*;*NFKB1*
Neurotrophin signaling pathway	12/119	5.66 × 10^−16^	2225.41	*NFKBIA*;*JUN*;*MAPK8*;*BAD*;*BCL2*;*BAX*;*AKT1*; *MAPK1*;*MAPK14*;*RAF1*;*RELA*;*NFKB1*
Relaxin signaling pathway	12/129	1.44 × 10^−15^	1979.36	*NFKBIA*;*JUN*;*MAPK8*;*TGFB1*;*NOS2*;*AKT1*; *MAPK1*;*MAPK14*;*RAF1*;*MMP9*;*RELA*;*NFKB1*
FoxO signaling pathway	12/131	1.70 × 10^−15^	1935.66	*IL10*;*IL6*;*CDKN1A*;*MAPK8*;*TGFB1*;*CCND1*; *CAT*;*AKT1*;*MAPK1*;*MAPK14*;*RAF1*;*SIRT1*
T cell receptor signaling pathway	11/104	6.02 × 10^−15^	2130.15	*IL10*;*NFKBIA*;*JUN*;*MAPK8*;*AKT1*;*MAPK1*; *MAPK14*;*RAF1*;*TNF*;*RELA*;*NFKB1*
Sphingolipid signaling pathway	11/119	2.70 × 10^−14^	1751.70	*MAPK8*;*PRKCB*;*BCL2*;*BAX*;*AKT1*; *MAPK1*;*MAPK14*;*RAF1*;*TNF*;*RELA*;*NFKB1*
Osteoclast differentiation	11/127	5.34 × 10^−14^	1593.97	*NFKBIA*;*JUN*;*MAPK8*;*TGFB1*;*IL1B*;*AKT1*; *MAPK1*;*MAPK14*;*TNF*;*RELA*;*NFKB1*
NOD-like receptor signaling pathway	12/181	7.10 × 10^−14^	1209.95	*NFKBIA*;*JUN*;*IL6*;*MAPK8*;*CASP8*;*IL1B*; *BCL2*;*MAPK1*;*MAPK14*;*TNF*;*RELA*;*NFKB1*
Prolactin signaling pathway	9/70	3.81 × 10^−13^	2208.04	*MAPK8*;*CCND1*;*AKT1*;*MAPK1*;*MAPK14*;*RAF1*;*ESR1*;*RELA*;*NFKB1*

**Table 3:** Representative ingredients of candidate herbs and its association with postmenopausal osteoporosis.

Name	Pubchem CID	Correlation Score	Overlap (*p*-Value ^#^)	References (PMID)
Benincasae Semen				
Rutin	5280805	0.0217	5/45 (3.76 × 10^−12^)	31737218
β-Sitosterol	222284	0.0161	1/11 (6.62 × 10^−3^)	35648689
Quercetin	5280343	0.0092	5/424 (3.43 × 10^−7^)	38240215
Glehniae Radix				
Falcarindiol *	5281148	0.0557	3/8 (5.91 × 10^−9^)	-
Corydalis Tuber				
Tetrahydrocoptisine *	6770	0.0846	3/5 (1.06 × 10^−6^)	-
Houttuyniae Herba				
Norisoboldine	14539911	0.0473	3/10 (1.26 × 10^−8^)	38813717
Hyperoside	5281643	0.0261	3/20 (1.20 × 10^−7^)	37157916
Rutin	5280805	0.0217	5/45 (3.76 × 10^−12^)	28485786
Quercetin	5280343	0.0092	5/424 (3.43 × 10^−7^)	34592982

The ^#^ denotes *p*-values calculated via the hypergeometric test to assess dataset overlap significance. * indicates active ingredients with strong associations to postmenopausal osteoporosis that remain uninvestigated.

## Data Availability

The data supporting the findings of this study are included within the manuscript.

## References

[1] Salari N., Ghasemi H., Mohammadi L., Behzadi M.H., Rabieenia E., Shohaimi S., Mohammadi M. (2021). The global prevalence of osteoporosis in the world: A comprehensive systematic review and meta-analysis. J. Orthop. Surg. Res..

[2] Eastell R., O’Neill T.W., Hofbauer L.C., Langdahl B., Reid I.R., Gold D.T., Cummings S.R. (2016). Postmenopausal osteoporosis. Nat. Rev. Dis. Primers.

[3] Reginster J.Y. (2011). Antifracture efficacy of currently available therapies for postmenopausal osteoporosis. Drugs.

[4] Adejuyigbe B., Kallini J., Chiou D., Kallini J.R. (2023). Osteoporosis: Molecular Pathology, Diagnostics, and Therapeutics. Int. J. Mol. Sci..

[5] Kennel K.A., Drake M.T. (2009). Adverse effects of bisphosphonates: Implications for osteoporosis management. Mayo Clin. Proc..

[6] Zhang X., Wang Z., Zhang D., Ye D., Zhou Y., Qin J., Zhang Y. (2023). The prevalence and treatment rate trends of osteoporosis in postmenopausal women. PLoS ONE.

[7] Lin J., Zhu J., Wang Y., Zhang N., Gober H.J., Qiu X., Li D., Wang L. (2017). Chinese single herbs and active ingredients for postmenopausal osteoporosis: From preclinical evidence to action mechanism. Biosci. Trends.

[8] Słupski W., Jawień P., Nowak B. (2021). Botanicals in Postmenopausal Osteoporosis. Nutrients.

[9] Wuttke W., Seidlová-Wuttke D., Gorkow C. (2003). The *Cimicifuga* preparation BNO 1055 vs. conjugated estrogens in a double-blind placebo-controlled study: Effects on menopause symptoms and bone markers. Maturitas.

[10] Wuttke W., Gorkow C., Seidlová-Wuttke D. (2006). Effects of black cohosh (*Cimicifuga racemosa*) on bone turnover, vaginal mucosa, and various blood parameters in postmenopausal women: A double-blind, placebo-controlled, and conjugated estrogens-controlled study. Menopause.

[11] García-Pérez M.A., Pineda B., Hermenegildo C., Tarín J.J., Cano A. (2009). Isopropanolic *Cimicifuga racemosa* is favorable on bone markers but neutral on an osteoblastic cell line. Fertil. Steril..

[12] Zhang Y., Li Q., Wan H.Y., Xiao H.H., Lai W.P., Yao X.S., Wong M.S. (2011). Study of the mechanisms by which *Sambucus williamsii* HANCE extract exert protective effects against ovariectomy-induced osteoporosis in vivo. Osteoporos. Int..

[13] Xiao H.H., Sham T.T., Chan C.O., Li M.H., Chen X., Wu Q.C., Mok D.K., Yao X.S., Wong M.S. (2018). A Metabolomics Study on the Bone Protective Effects of a Lignan-Rich Fraction From *Sambucus Williamsii* Ramulus in Aged Rats. Front. Pharmacol..

[14] Chae H.J., Chae S.W., Yun D.H., Keum K.S., Yoo S.K., Kim H.R. (2004). Prevention of bone loss in ovariectomized rats: The effect of *Salvia miltiorrhiza* extracts. Immunopharmacol. Immunotoxicol..

[15] Guo Y., Li Y., Xue L., Severino R.P., Gao S., Niu J., Qin L.P., Zhang D., Brömme D. (2014). *Salvia miltiorrhiza*: An ancient Chinese herbal medicine as a source for anti-osteoporotic drugs. J. Ethnopharmacol..

[16] Sudarshan K., Yarlagadda S., Sengupta S. (2024). Recent Advances in the Synthesis of Diarylheptanoids. Chem. Asian J..

[17] Noor F., Tahir Ul Qamar M., Ashfaq U.A., Albutti A., Alwashmi A.S.S., Aljasir M.A. (2022). Network Pharmacology Approach for Medicinal Plants: Review and Assessment. Pharmaceuticals.

[18] Ge Q., Chen L., Tang M., Zhang S., Liu L., Gao L., Ma S., Kong M., Yao Q., Feng F. (2018). Analysis of mulberry leaf components in the treatment of diabetes using network pharmacology. Eur. J. Pharmacol..

[19] Luo T., Zhao Z.H., Wu M.R., Ren X.Y., Xu Z.Y., Li L.J., Yi Y., Wang H.X., Wang L.M. (2024). Network pharmacology screening, in vitro and in vivo evaluation of antianxiety and antidepressant drug-food analogue. Phytomedicine.

[20] Ruiz C., Zitnik M., Leskovec J. (2021). Identification of disease treatment mechanisms through the multiscale interactome. Nat. Commun..

[21] Sugiyama M.G., Cui H., Redka D.S., Karimzadeh M., Rujas E., Maan H., Hayat S., Cheung K., Misra R., McPhee J.B. (2021). Multiscale interactome analysis coupled with off-target drug predictions reveals drug repurposing candidates for human coronavirus disease. Sci. Rep..

[22] Lin H., Gao Y.F., Wei C., Wang M.L., Ma X.H. (2024). Effects of *Sophora japonica* extract on alveolar bone mass in ovariectomized osteoporosis mice. Shanghai Kou Qiang Yi Xue.

[23] Zhao X., Mei L., Pei J., Liu Z., Shao Y., Tao Y., Zhang X., Jiang L. (2017). Sophoridine from Sophora Flower Attenuates Ovariectomy Induced Osteoporosis through the RANKL-ERK-NFAT Pathway. J. Agric. Food Chem..

[24] Tran P.T., Park D.H., Kim O., Kwon S.H., Min B.S., Lee J.H. (2018). Desoxyrhapontigenin inhibits RANKL induced osteoclast formation and prevents inflammation mediated bone loss. Int. J. Mol. Med..

[25] Kim J.H., Kim M., Jung H.S., Sohn Y. (2019). *Leonurus sibiricus* L. Ethanol extract promotes osteoblast differentiation and inhibits osteoclast formation. Int. J. Mol. Med..

[26] Zhao B., Peng Q., Poon E.H.L., Chen F., Zhou R., Shang G., Wang D., Xu Y., Wang R., Qi S. (2021). Leonurine Promotes the Osteoblast Differentiation of Rat BMSCs by Activation of Autophagy via the PI3K/Akt/mTOR Pathway. Front. Bioeng. Biotechnol..

[27] Kim J.Y., Baek J.M., Ahn S.J., Cheon Y.H., Park S.H., Yang M., Choi M.K., Oh J. (2016). Ethanolic extract of *Schizonepeta tenuifolia* attenuates osteoclast formation and activation in vitro and protects against lipopolysaccharide-induced bone loss in vivo. BMC Complement. Altern. Med..

[28] Xu T., Yin J., Dai X., Liu T., Shi H., Zhang Y., Wang S., Yue G., Zhang Y., Zhao D. (2024). Cnidii Fructus: A traditional Chinese medicine herb and source of antiosteoporotic drugs. Phytomedicine.

[29] Ma Y., Wang L., Zheng S., Xu J., Pan Y., Tu P., Sun J., Guo Y. (2019). Osthole inhibits osteoclasts formation and bone resorption by regulating NF-κB signaling and NFATc1 activations stimulated by RANKL. J. Cell Biochem..

[30] Nian H., Qin L.P., Chen W.S., Zhang Q.Y., Zheng H.C., Wang Y. (2006). Protective effect of steroidal saponins from rhizome of *Anemarrhena asphodeloides* on ovariectomy-induced bone loss in rats. Acta Pharmacol. Sin..

[31] Kim Y.W., Bak S.B., Song Y.R., Kim C.E., Lee W.Y. (2024). Systematic exploration of therapeutic effects and key mechanisms of *Panax ginseng* using network-based approaches. J. Ginseng Res..

[32] Han S.Y., Kim J.H., Bae G.S., Lee W.Y. (2024). Identifying Candidate Polyphenols Beneficial for Oxidative Liver Injury through Multiscale Network Analysis. Curr. Issues Mol. Biol..

[33] Katayama Y., Akatsu T., Yamamoto M., Kugai N., Nagata N. (1996). Role of nonenzymatic glycosylation of type I collagen in diabetic osteopenia. J. Bone Miner. Res..

[34] Li Z., Li C., Zhou Y., Chen W., Luo G., Zhang Z., Wang H., Zhang Y., Xu D., Sheng P. (2016). Advanced glycation end products biphasically modulate bone resorption in osteoclast-like cells. Am. J. Physiol. Endocrinol. Metab..

[35] Sancho D., Reis e Sousa C. (2012). Signaling by myeloid C-type lectin receptors in immunity and homeostasis. Annu. Rev. Immunol..

[36] Fischer V., Haffner-Luntzer M. (2022). Interaction between bone and immune cells: Implications for postmenopausal osteoporosis. Semin. Cell Dev. Biol..

[37] Rached M.T., Kode A., Xu L., Yoshikawa Y., Paik J.H., Depinho R.A., Kousteni S. (2010). FoxO1 is a positive regulator of bone formation by favoring protein synthesis and resistance to oxidative stress in osteoblasts. Cell Metab..

[38] Qi T., Li L., Weidong T. (2021). The Role of Sphingolipid Metabolism in Bone Remodeling. Front. Cell Dev. Biol..

[39] Zhu C., Shen S., Zhang S., Huang M., Zhang L., Chen X. (2022). Autophagy in Bone Remodeling: A Regulator of Oxidative Stress. Front. Endocrinol..

[40] Lotinun S., Westerlind K.C., Kennedy A.M., Turner R.T. (2003). Comparative effects of long-term continuous release of 16 alpha-hydroxyestrone and 17 beta-estradiol on bone, uterus, and serum cholesterol in ovariectomized adult rats. Bone.

[41] Lim S.K., Won Y.J., Lee J.H., Kwon S.H., Lee E.J., Kim K.R., Lee H.C., Huh K.B., Chung B.C. (1997). Altered hydroxylation of estrogen in patients with postmenopausal osteopenia. J. Clin. Endocrinol. Metab..

[42] Islam M.T., Quispe C., El-Kersh D.M., Shill M.C., Bhardwaj K., Bhardwaj P., Sharifi-Rad J., Martorell M., Hossain R., Al-Harrasi A. (2021). A Literature-Based Update on *Benincasa hispida* (Thunb.) Cogn.: Traditional Uses, Nutraceutical, and Phytopharmacological Profiles. Oxid. Med. Cell Longev..

[43] Li S., Xu N., Fang Q., Cheng X., Chen J., Liu P., Li L., Wang C., Liu W. (2023). *Glehnia littoralis* Fr. Schmidtex Miq.: A systematic review on ethnopharmacology, chemical composition, pharmacology and quality control. J. Ethnopharmacol..

[44] Alhassen L., Dabbous T., Ha A., Dang L.H.L., Civelli O. (2021). The Analgesic Properties of *Corydalis yanhusuo*. Molecules.

[45] Wei P., Luo Q., Hou Y., Zhao F., Li F., Meng Q. (2024). *Houttuynia Cordata* Thunb.: A comprehensive review of traditional applications, phytochemistry, pharmacology and safety. Phytomedicine.

[46] Charachit N., Sukhamwang A., Dejkriengkraikul P., Yodkeeree S. (2022). Hyperoside and Quercitrin in *Houttuynia cordata* Extract Attenuate UVB-Induced Human Keratinocyte Cell Damage and Oxidative Stress via Modulation of MAPKs and Akt Signaling Pathway. Antioxidants.

[47] Zanotti S., Stadmeyer L., Smerdel-Ramoya A., Durant D., Canalis E. (2009). Misexpression of CCAAT/enhancer binding protein beta causes osteopenia. J. Endocrinol..

[48] Thielen N.G.M., van der Kraan P.M., van Caam A.P.M. (2019). TGFβ/BMP Signaling Pathway in Cartilage Homeostasis. Cells.

[49] Sun J., Zhang C., Xu L., Yang M., Yang H. (2015). The transforming growth factor-β1 (TGF-β1) gene polymorphisms (TGF-β1 T869C and TGF-β1 T29C) and susceptibility to postmenopausal osteoporosis: A meta-analysis. Medicine.

[50] Hughes D.E., Dai A., Tiffee J.C., Li H.H., Mundy G.R., Boyce B.F. (1996). Estrogen promotes apoptosis of murine osteoclasts mediated by TGF-beta. Nat. Med..

[51] Zou J., Qiu Z.C., Yu Q.Q., Wu J.M., Wang Y.H., Shi K.D., Li Y.F., He R.R., Qin L., Yao X.S. (2024). Discovery of a Potent Antiosteoporotic Drug Molecular Scaffold Derived from *Angelica sinensis* and Its Bioinspired Total Synthesis. ACS Cent. Sci..

[52] Wishart D.S., Feunang Y.D., Guo A.C., Lo E.J., Marcu A., Grant J.R., Sajed T., Johnson D., Li C., Sayeeda Z. (2018). DrugBank 5.0: A major update to the DrugBank database for 2018. Nucleic Acids Res..

[53] Wang Y., Zhang S., Li F., Zhou Y., Zhang Y., Wang Z., Zhang R., Zhu J., Ren Y., Tan Y. (2020). Therapeutic target database 2020: Enriched resource for facilitating research and early development of targeted therapeutics. Nucleic Acids Res..

[54] Szklarczyk D., Santos A., von Mering C., Jensen L.J., Bork P., Kuhn M. (2016). STITCH 5: Augmenting protein-chemical interaction networks with tissue and affinity data. Nucleic Acids Res..

[55] Koopmans F., van Nierop P., Andres-Alonso M., Byrnes A., Cijsouw T., Coba M.P., Cornelisse L.N., Farrell R.J., Goldschmidt H.L., Howrigan D.P. (2019). SynGO: An Evidence-Based, Expert-Curated Knowledge Base for the Synapse. Neuron.

[56] Kuleshov M.V., Jones M.R., Rouillard A.D., Fernandez N.F., Duan Q., Wang Z., Koplev S., Jenkins S.L., Jagodnik K.M., Lachmann A. (2016). Enrichr: A comprehensive gene set enrichment analysis web server 2016 update. Nucleic Acids Res..

[57] Fang Z., Liu X., Peltz G. (2023). GSEApy: A comprehensive package for performing gene set enrichment analysis in Python. Bioinformatics.

[58] Gene Ontology Consortium (2015). Gene Ontology Consortium: Going forward. Nucleic Acids Res..

[59] Kanehisa M., Goto S., Sato Y., Kawashima M., Furumichi M., Tanabe M. (2014). Data, information, knowledge and principle: Back to metabolism in KEGG. Nucleic Acids Res..

[60] Piñero J., Ramírez-Anguita J.M., Saüch-Pitarch J., Ronzano F., Centeno E., Sanz F., Furlong L.I. (2020). The DisGeNET knowledge platform for disease genomics: 2019 update. Nucleic Acids Res..

